# Educational inequalities in patient-centred care: patients' preferences and experiences

**DOI:** 10.1186/1472-6963-12-261

**Published:** 2012-08-17

**Authors:** Jany Rademakers, Diana Delnoij, Jessica Nijman, Dolf de Boer

**Affiliations:** 1 NIVEL (Netherlands Institute for Health Services Research), PO Box 1568, 3500, BN, Utrecht, The Netherlands; 2 CKZ (Centre for Consumer Experiences in Healthcare), Utrecht, the Netherlands; 3 Scientific Centre for Transformation in Care and Welfare (Tranzo), Tilburg University, Tilburg, The Netherlands

**Keywords:** Patient preferences, Patient experiences, Communication, Information, Shared decision making, Education, Inequalities, Patient-centred care

## Abstract

**Background:**

Educational attainment is strongly related to specific health outcomes. The pathway in which individual patient-provider interactions contribute to (re)producing these inequalities has yet to be studied. In this article, the focus is on differences between less and more highly educated patients in their preferences for and experiences with patient-centred care., e.g. shared decision making, receiving understandable explanations and being able to ask questions.

**Methods:**

Data are derived from several Consumer Quality-index (CQ-index) studies. The CQ-index is a family of standardized instruments which are used in the Netherlands to measure quality of care from the patient’s perspective.

**Results:**

The educational level of patients is directly related to the degree of importance patients attribute to specific aspects of patient-centred care. It has a minor influence on the experienced level of shared decision making, but not on experiences regarding other aspects of patient-centred care.

**Conclusions:**

All patients regard patient-centred care as important and report positive experiences. However, there is a discrepancy between patient preferences for patient-centred care on one hand and the care received on the other. Less educated patients might receive ‘too much’, and more highly educated patients ‘too little’ in the domains of communication, information and shared decision making.

## Background

Educational inequalities in health outcomes have consistently been reported in all western countries over the last decades [1-4]. In general, educational attainment is strongly and inversely related to specific health indicators, such as mortality, incidence of cancer, cardiovascular diseases, chronic illnesses such as diabetes and asthma/COPD, and subjective health. However, the way in which education influences health is still an object of study. Several mediating variables have been researched, including lifestyle and health behaviours (e.g. smoking, diet, exercising), work, economic and environmental factors, social-psychological resources [5], health literacy [6,7] and access to health care [8,9]. The pathway in which individual patient-provider interactions in health care contribute to (re)producing educational inequalities has been less studied. Nevertheless, the American Institute of Medicine states that understanding and improving communication may be a key to addressing disparities in health outcomes [10]. In this research area the main questions would be: is the health care provider able to provide personalized, tailored services that meet the needs of less educated patients, and are less educated patients willing and able to communicate with their health care provider and to participate in the care process? Both questions touch upon the domain of patient-centred care. Patient-centred care represents a humanistic, bio-psychosocial perspective in health care. It puts a relatively strong emphasis on communication and information and takes the patient’s perspective as a starting point, thereby tuning medical care to the individual patient’s needs and preferences. Furthermore patient-centred care focuses on patient participation in clinical decision making, forming a therapeutic alliance and sharing power and responsibility. Patient-centred care is often positioned as the opposite of a biomedical, disease-oriented, evidence-based medical approach [11,12].

Many studies demonstrate that providing patient-centred, tailored care has a positive influence on different health outcomes [13], e.g. mortality [14], health behaviour [15], treatment adherence [16], and self management [17]. Not all studies on the effect of patient-centred care report a positive influence, which may be due to different definitions, different conditions, or different patient groups with varying preferences [18]. Though most patients value a patient-centred approach, there is evidence that especially less educated patients are less responsive to it [13,19]. Whereas the majority of patients prefer a patient-centred communication style, less educated patients are more likely to prefer a directive, biomedical approach [13,18]. Furthermore, less educated patients are found to prefer a more passive role in decision making [20].

Whatever the preferences of less educated patients are with respect to the communication style of their provider, it is generally agreed upon that every patient has the right to good quality of care. Some studies, however, have demonstrated a negative impact of a lesser educational level on the quality of the patient-provider communication [21]. In this article, we shall further explore this topic, focusing on what patients find important and what their actual experiences are in the consultation room.

The following research questions will be addressed:

1. Are there any differences between less and more highly educated patients in the importance they attribute to aspects of patient-centred care?

2. Are there any differences between the experiences of less and more highly educated patients regarding aspects of patient-centred care?

The aspects of patient-centred care that will be studied are: shared decision making, receiving understandable explanations and being able to ask questions.

## Methods

### Consumer Quality-index

The data for this study were derived from Consumer Quality-index (CQ-index or CQI) development studies. The CQ-index is a family of standardized instruments which are used in the Netherlands to measure quality of care from the patient’s perspective. Both the content of the questionnaires and the way in which data are collected and analyzed are standardized to ensure the possibility of generating comparative information (between providers, between patient groups, between certain periods in time). There are CQ-index questionnaires for specific sectors in health care (e.g. hospital care), for procedures (e.g. hip- and knee surgery), and for patients groups (e.g. patients with rheumatoid arthritis, asthma/COPD, spinal disc herniation etc.). With a CQ-index patients’ experiences are measured as well as their priorities. The content of a CQ-index questionnaire typically consists of questions regarding the frequency with which quality criteria were met (never, sometimes, usually, always) and the extent to which performance on quality criteria has raised problems (big problem, small problem, no problem). In addition, respondents are requested to provide global ratings on (elements of) the care received using a 10-point Likert scale. In addition, a number of standard patient characteristics are assessed in all CQ-index surveys, such as age, sex and educational level as well as questions regarding disease-specific patient characteristics. Finally, questions regarding the importance attributed to certain aspects of health care are posed (not really important, of some importance, of substantial importance, of the utmost importance). Since importance scores are generally constant they are not routinely examined in CQ-index surveys, but assessed during its development and replicated when deemed appropriate. The possibility of combining patient experiences and importance scores is a unique feature of the CQ-index. This enables users of the instrument (care providers, health insurers, patient organizations, policy makers, researchers) to locate precisely the aspects of care where the need for quality improvement is most (or least) necessary from the perspective of the individual patient or specific patient groups.

In all CQ-index questionnaires, some questions are routinely asked about the patient-provider interaction. For this study, we have used two questions which reflect aspects of quality of communication (‘Did the doctor give you understandable explanations about your disease and the treatment?’ and ‘Were you able to ask the doctor any questions if you wanted to?’) and one question on shared decision making (‘Were you able to participate in decisions about your treatment or care?’). We chose these items because of two reasons. Firstly, communication is the means to actually demonstrate a patient-centred approach, with a focus on the personal needs and questions of the patient. Secondly, shared responsibility is a goal of patient-centred care, in which the patient and the doctor are regarded as partners in the therapeutic process [11,12].

### Participants and demographic characteristics

For the purpose of this present study, data from three CQ-index development studies were selected: CQI Rheumatoid arthritis, CQI Spinal disc herniation and CQI Breast care (patients with both malignant and benign breast abnormalities). The original development studies have been published in Dutch reports [22-24]. These groups were selected because we aimed to cover a broad spectrum of patients, since the importance of and experiences with patient-centred care may be different in specific situations. By choosing these three groups there was a variety in our dataset by type of disease (relative acute to chronic) and treatments (surgical, pharmaceutical and conservative). Furthermore, the availability of the data facilitated the choice for this selection.

All data were collected in the Netherlands. The data collection procedure for CQ-index studies always follows a standard protocol. Patients were recruited through insurance companies and/or hospitals, and were approached by mail using a procedure known as the Dillman method [25], which includes up to four mail shots if necessary. Patients received both a survey on their experiences with the care they had received and on their priorities. CQ-index studies do not fall within the scope of the Dutch Medical Research Involving Human Subjects Act and therefore neither medical approval for the original studies nor for these secondary analyses was required.

The dataset for patients suffering from rheumatoid arthritis consisted of 349 patients (response = 59.6 %), the dataset for patients suffering from spinal disc herniation contained 145 patients (response = 37.1 %) and the dataset for patients suffering from malignant or benign breast abnormalities consisted of 515 patients (response = 50.4 %). The demographic characteristics of the patient groups are listed in Table 1.

**Table 1:** Patient characteristics

**Group**	**N**	**gender**	**age**			**education**^**1**^		
		**male**	**<45**	**45-65**	**>65**	**low**	**medium**	**high**
Benign breast abnormality	212	0,0 %	32,1 %	56,1 %	11,8 %	29,2 %	37,3 %	33,5 %
Malignant breast abnormality	313	0,0 %	11,2 %	54,3 %	34,5 %	37,7 %	39,9 %	22,4 %
Rheumatoid arthritis	349	26,6 %	9,7 %	47,0 %	43,3 %	48,4 %	35,0 %	16,6 %
Spinal disc herniation	145	53,1 %	25,5 %	49,7 %	24,8 %	33,8 %	37,9 %	28,3 %

^1^ Any education up to lower secondary education was coded as ‘low’, upper secondary education was coded as ‘medium’ and both post secondary non-tertiary education and tertiary education were coded as ‘high’.

### Statistical analysis and graphical representation

For ease of graphical representation, both experience and importance scores were plotted as means with standard errors using bar charts for each educational level. For formal statistical testing, the ordinal nature of the dependent variables was accommodated using proportional odds regression. The dependent variables were the importance and experiences scores and the independent variables were patient group, gender, age and education. For each independent variable, two models were fitted: a model including all independent variables except for education (model A) and a subsequent model in which education was added as an explanatory variable (model B). Differences between the models were tested with the Likelihood Ratio test (LR). All analyses were conducted using STATA 10.0.

## Results

### Importance

**Figure 1:**
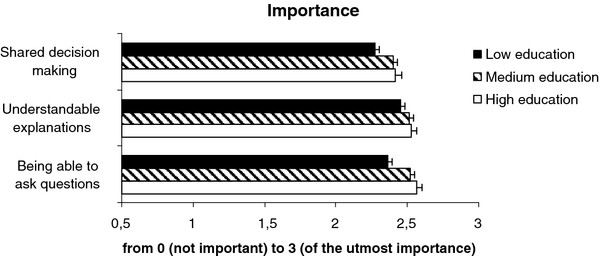
Importance.

The educational level of patients was directly related to the degree of importance they attributed to specific aspects of patient-centred care, e.g. shared decision making, receiving understandable explanations and being able to ask questions (Figure 1). The differences between the three groups, however, were small: the mean scores (on a 0 to 3 scale) varied from 2.3 to 2.6. This implies that for all groups patient-centred care was very important.

In Table 2 a comparison of two regression models (model A including patient group, gender and age; model B including the aforementioned characteristics plus educational level) demonstrated that educational level in itself contributes to the explanation of differences with respect to importance scores. Lower educated patients found these aspects of patient-centred care less important compared with higher educated patients (OR’s 0.53 – 0.84 for low vs. high education; OR’s 0.83 – 0.95 for medium vs. high education). With respect to shared decision making and being able to ask questions, the difference between lowest and highest educated patients was significant, for receiving understandable explanations and patients with a medium education level the trend -although not significant - was in the same direction (see Table 2).

**Table 2:** Odds Ratio models by patient group, gender, age and education

	**Importance**			**Experience**		
	**OR's model a**	**OR's model b**	**LR test**	**OR's model a**	**OR's model b**	**LR test**
*Shared decision making*			*p* < 0.05			*p* < 0.05
…Benign breast abnormality	reference	reference		reference	reference	
…Malignant breast …abnormality	1,03	1,02		1,28	1,49	
…Reumatoid arthritis	0,87	0,87		1,54	1,75	
…Spinal disc herniation	1,46	1,39		1,38	1,65	
…Female	reference	reference		reference	reference	
…Male	**0,48**	**0,50**		1,01	0,96	
…Age < 45	reference	reference		reference	reference	
…Age 45-65	**1,71**	**1,77**		1,17	1,14	
…Age > 65	1,45	**1,59**		1,18	1,07	
…High education	reference	reference		reference	reference	
…Medium education	-	0,92		-	0,83	
…Low education	-	**0,67**		-	1,34	
*Understandable explanations*^*1*^			n.s.			n.s.
…Benign breast abnormality	reference	reference		reference	reference	
…Malignant breast …abnormality	0,77	0,77		1,08	1,09	
…Reumatoid arthritis	**0,37**	**0,38**		0,95	0,96	
…Spinal disc herniation	**0,56**	**0,56**		1,11	1,11	
…Female	reference	reference		reference	reference	
…Male	**0,65**	**0,66**		**1,66**	**1,64**	
…Age < 45	reference	reference		reference	reference	
…Age 45-65	**1,95**	**1,97**		**1,43**	**1,42**	
…Age > 65	**1,55**	**1,61**		1,28	1,26	
…High education	reference	reference		reference	reference	
…Medium education	-	0,95		-	0,90	
…Low education	-	0,84		-	1,00	
*Being able to ask questions*^*2*^			*p* < 0.05			n.s.
…Benign breast abnormality	reference	reference		reference	reference	
…Malignant breast …abnormality	0,91	0,93		**1,86**	**1,85**	
…Reumatoid arthritis	**0,57**	**0,60**		**2,06**	**2,03**	
…Spinal disc herniation	**0,62**	**0,61**		1,13	1,14	
…Female	reference	reference		reference	reference	
…Male	**0,59**	**0,63**		1,59	1,56	
…Age < 45	reference	reference		reference	reference	
…Age 45-65	1,40	**1,47**		1,44	1,43	
…Age > 65	0,92	1,07		1,27	1,22	
…High education	reference	reference		reference	reference	
…Medium education	-	0,83		-	1,06	
…Low education	-	**0,53**		-	1,19	

significant results (p < 0.05) are **bold**.

model a: patient group, gender and age.

model b: model a + education.

LR test: likelihood ratio test.

^1^ The proportional odds assumption was violated for the experience scores. A subsequent analysis that did not depend on the proportional odds assumption yielded virtually identical results regarding the transfer from 'usually' to 'always' and showed some deviations for the transfer from 'never / sometimes' to 'usually'.

^2^ The proportional odds assumption was violated for the importance scores. A subsequent analysis that did not depend on the proportional odds assumption yielded virtually identical results regarding the transfer from 'important' to 'of the utmost importance' and showed some deviations for the transfer from 'not (really) important' to 'important'.

This table also shows that male patients found all these aspects of patient-centred care significantly less important compared with female patients, regardless of their educational level. Patients above 45 years found shared decision making, receiving understandable explanations and (to a lesser extent) being able to ask questions more important compared with younger patients. There were differences between patient groups in the extent to which they regarded aspects of patient care important: patients with rheumatoid arthritis or spinal disc herniation found receiving understandable explanations and being able to ask questions less important compared with the other patient groups, but the patients with spinal disc herniation found it significantly more important to be involved in shared decision making. These effects seemed independent of educational level, as including education hardly affected the OR’s for patient groups.

### Experience

**Figure 2:**
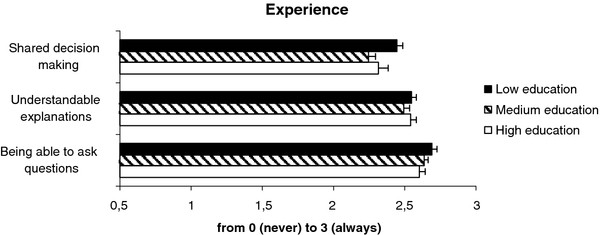
Experience.

A distinct relationship between the educational level of patients and their experiences in the consultation room regarding the three aspects of patient-centred care was absent (Figure 2). In general, the lowest educated patients reported most positive experiences with all aspects of patient-centred care, but the differences with the other groups were very small: for the aspect shared decision making scores vary between 2.3 (medium education level) and 2.4 (low), for understandable explanations between 2.4 (medium) and 2.5 (low), and for being able to ask questions between 2.6 (high) and 2.7 (low). As with importance the scores were very high: all patients mainly reported frequent experiences with these aspects of patient-centred care.

Comparing the regression models including and excluding education (Table 2) showed that educational level indeed did not add much to the model which included patient group, gender and age. It only contributed to the explanation of differences with respect to experiences regarding shared decision making, although the separate odds ratios for education did not reach statistical significance in this model. Moreover, the odds ratio for less education here was higher, indicating that patients in this group reported being more often involved in shared decision making (OR 1.34 for low vs. high education). The same trend was noticeable for being able to ask questions (OR 1.19 for low vs. high education).

Though the educational level of patients did not have much impact on their experiences with patient-centred care, gender and age did. Men and older patients (45-65 years) more often reported receiving understandable explanations and/or being able to ask questions. There also were significant differences between patient groups: patients with rheumatoid arthritis and with a malignant breast abnormality more often reported that they had been able to ask questions.

## Discussion

In general, our study shows that patients regard patient-centred care as important and that their experiences are positive. Both the reported importance and the experience scores are very high. Since all patients mainly reported frequent experiences with the three aspects of patient-centred care, and since there was very little variation by educational level in this respect, it can be concluded from our study that there are no educational inequalities with respect to the amount of patient-centred care patients receive.

Focusing on the differences between subgroups in our study, it is clear that patients with a low education level regarded all three aspects of patient-centred care as less important. This finding was strongest for aspects in which an active role is expected of the patient (shared decision making, being able to ask questions). Our study confirms the results of earlier studies [13,18,20] that less educated patients generally have a lower preference for a patient-centred communication style. However, the patients with a low education level in our study reported as much (or in the case of shared decision making and being able to ask questions even more) frequent experiences as patients with a high educational level. Viewed from the opposite perspective, patients with a higher education level attached more importance to these aspects, but their experiences were the same as for less educated patients. This discrepancy between patient preferences on one hand and the care received on the other might have a negative impact on patient satisfaction [26-29]. According to our study, less educated patients might receive ‘too much’ in the domains of communication, information and shared decision making, and high educated patients ‘too little’ (though with these high experience scores, there is not much room for improvement). Since patient satisfaction is in turn associated with specific health outcomes, such as self-perceived health [30] and mortality [31], a discrepancy between patient preferences and experiences in health care can negatively affect health. It should be noted though, that it is unclear to what extent the differences in experiences reported by these patient groups reflect actual differences in the care received. In the patient experience literature, it has often been described that patients with a lower level of education report more positive experiences than patients with a higher level of education [32,33]. This might reflect a systematic reporting bias rather than real differences in patients’ experiences [34].

Furthermore, instead of focusing on frequencies as is done in the CQ-index questionnaires -as in many other patient experience questionnaires- the content of the patient-provider interaction should be studied as well. Patients might report the same amount of positive experiences, but the way in which questions are asked and dealt with or information is presented and understood might be very different. In general, it is known that less educated patients more often lack anatomical knowledge [35], and have lower levels of health literacy [7]. This means that they will have more difficulty obtaining, processing and understanding basic health information needed to make appropriate health decisions (definition Health literacy American Institutes of Medicine; [36]). Apart from knowledge and functional literacy, other patient-related factors such as motivation, self-confidence and social skills will influence the patient’s role in the interaction [37]. These factors are combined in the concept of ‘patient activation’: the knowledge, skills and confidence to self-manage one’s health or chronic condition [38,39]. Higher patient activation scores on a validated measure correlate with better preventive and self-management behaviours and better health outcomes. However, patients with lower educational levels generally score lower on this instrument indicating that they feel less confident and are more likely to be passive recipients of care. Improving health literacy and activation of less educated patients will enhance their position in the medical interaction and thus the chance for better health outcomes.

On the part of the care provider, there is also room for improvement. The crucial counterpart of an empowered patient is a ‘patient literate’ health professional. Throughout different European countries, patients value doctors who provide space for patients to ask questions and express their concerns, as well as doctors who provide tailor-made communication [40]. [Edwards et al. 41] demonstrated that the degree to which the care provider is receptive to patients and patient choice and has knowledge of cultural differences positively influences information exchange and shared decision making in consultations. Stereotyping patients was found to have a negative impact on the interaction. In a study on the effects of patient-centred care, an activating and supporting communication style of the doctor proved to be most effective [18]. In those situations where doctors invited and encouraged patients to actively participate in the interaction, patients were more involved, whereas providers who tried to take the patient’s perspective in their communication had less positive results. It is possible that taking the patient’s perspective more easily leads to stereotyping (and thinking on behalf of) the patient.

This present study does have some limitations. In the sample, men are underrepresented as a consequence of including data from patients who suffered from benign or malignant breast abnormalities. Since education and not gender was the focus of our article, and education was well-distributed among the different patient groups, this is not likely to have affected our results. Furthermore, in spite of the fact that most response rates are within the normal range for patient experience questionnaires, the response rate of the patients with spinal disc herniation was low (37.1 %). This sample might therefore not be representative of the population from which it is drawn. A very important limitation has already been mentioned in the discussion section, namely that there might be a systematic reporting bias, in the sense that patients with a lower level of education report more positive experiences than patients with a higher level of education. Since the education level was our main independent variable, we could not correct our data in this respect as is done, for example, in comparative studies (case-mix adjustment). This could mean that our conclusions are too optimistic with respect to the patient-centred care experiences of lower educated patients. Another systematic bias is the fact that less educated, and therefore usually less literate patients will have more problems filling out a self-administered questionnaire. With respect to CQ-index questionnaires for example, the item non-response among patients from non-western origin is known to be larger compared to Dutch born patients [42]. In general, non respondents of patient experience questionnaires are more likely to be illiterate [43]. Therefore the less educated patients in our sample will represent the most literate, which again may have a positive impact on our conclusions. The last limitation refers to the validity of the way in which we have operationalized patient-centred care. Both the aspects under study (shared decision making, receiving understandable explanations, being able to ask questions) and the particular quantitative research method utilized here (measuring importance scores and frequencies of experiences) only reflect parts of this broad concept. To fully understand the processes in which educational level impacts health outcomes, more qualitative, in depth information on the patient-provider interaction and its determinants is necessary.

## Conclusions

In this article two research questions were addressed, focusing on the differences between less and more highly educated patients in (1) the importance they attribute to specific aspects of patient-centred care, e.g. shared decision making, receiving understandable explanations and being able to ask questions, and (2) the experiences they report with respect to these aspects. The educational level of patients is directly related to the degree of importance they attribute to specific aspects of patient-centred care: lower educated patients generally found these aspects less important compared with higher educated patients. The reported experiences with patient-centred care, however, were similar for more highly and less educated patients.

## Competing interests

The authors declare they have no competing interests.

## Authors' contributions

JR conceived and designed the study and wrote the first draft of the manuscript, DdB contributed to the conception and design of the study, performed the data-analysis and contributed to the first draft. JN prepared the dataset and assisted DdB in the data-analysis. DdB and DD commented on and contributed to draft versions of the manuscript. All authors read and approved the final manuscript.

## Pre-publication history

The pre-publication history for this paper can be accessed here:

http://www.biomedcentral.com/1472-6963/12/261/prepub
